# A network-based explanation of why most COVID-19 infection curves are linear

**DOI:** 10.1073/pnas.2010398117

**Published:** 2020-08-24

**Authors:** Stefan Thurner, Peter Klimek, Rudolf Hanel

**Affiliations:** ^a^Section for Science of Complex Systems, Center for Medical Statistics, Informatics and Intelligent Systems, Medical University of Vienna, A-1090 Vienna, Austria;; ^b^Complexity Science Hub Vienna, A-1080 Vienna, Austria;; ^c^Santa Fe Institute, Santa Fe, NM 85701

**Keywords:** compartmental epidemiological model, mean-field (well mixed) approximation, social contact networks, network theory, COVID-19

## Abstract

Many countries have passed their first COVID-19 epidemic peak. Traditional epidemiological models describe this as a result of nonpharmaceutical interventions pushing the growth rate below the recovery rate. In this phase of the pandemic many countries showed an almost linear growth of confirmed cases for extended time periods. This new containment regime is hard to explain by traditional models where either infection numbers grow explosively until herd immunity is reached or the epidemic is completely suppressed. Here we offer an explanation of this puzzling observation based on the structure of contact networks. We show that for any given transmission rate there exists a critical number of social contacts, $D_{c}$, below which linear growth and low infection prevalence must occur. Above $D_{c}$ traditional epidemiological dynamics take place, e.g., as in susceptible–infected–recovered (SIR) models. When calibrating our model to empirical estimates of the transmission rate and the number of days being contagious, we find $D_{c}∼7.2$. Assuming realistic contact networks with a degree of about 5, and assuming that lockdown measures would reduce that to household size (about 2.5), we reproduce actual infection curves with remarkable precision, without fitting or fine-tuning of parameters. In particular, we compare the United States and Austria, as examples for one country that initially did not impose measures and one that responded with a severe lockdown early on. Our findings question the applicability of standard compartmental models to describe the COVID-19 containment phase. The probability to observe linear growth in these is practically zero.

## Significance

For many countries a plain-eye inspection of the COVID-19 infection curves reveals a remarkable linear growth over extended time periods. This observation is practically impossible to understand with traditional epidemiological models. These, to make them expressible in compact mathematical form, typically ignore the structure of real contact networks that are essential in the characteristic spreading dynamics of COVID-19. Here we show that by properly taking some relevant network features into account, linear growth can be naturally explained. Further, the effect of nonpharmaceutical interventions (NPIs), like national lockdowns, can be modeled with a remarkable degree of precision without fitting or fine-tuning of parameters.

Textbook knowledge of epidemiology has it that an epidemic event comes to a halt when herd immunity in a population is reached (1, 2). Herd immunity levels depend on the disease. For influenza it is within the range of 33 to 44% of the population (3), for Ebola it is 33 to 60% (4), for measles it is 92 to 95% (5), and for the Severe Acute Respiratory Syndrome (SARS) levels between 50 and 80% are reported (6). For the current COVID-19 outbreak it is expected to be in the range of 29 to 74% (7, 8). On the way toward herd immunity, textbook knowledge teaches, the number of infected increases faster than linear (in early phases even exponentially) as long as the effective reproduction number is larger than 1. Once this threshold is passed, the daily increments in the number of infected start to decrease until they drop to zero (1, 9). Combining these two growth phases yields the characteristic S-shaped infection curves.

The COVID-19 outbreak shows a very different picture, however. Several countries have clearly passed a first maximum of the epidemic and are converging toward zero new cases per day. None of these countries are even close to herd immunity. In Austria at the first peak of the pandemic so far, a population-wide representative PCR study showed that only about 0.3% of the population tested positive (10). Similarly, in Iceland in a random-population screening the prevalence of positively tested was found to be 0.8% (11). Clearly, at this time the COVID-19 outbreak has been far from the uncontrolled case as many countries have implemented nonpharmaceutical interventions (NPIs) to reduce infection rates (12).

**Fig. 1.**
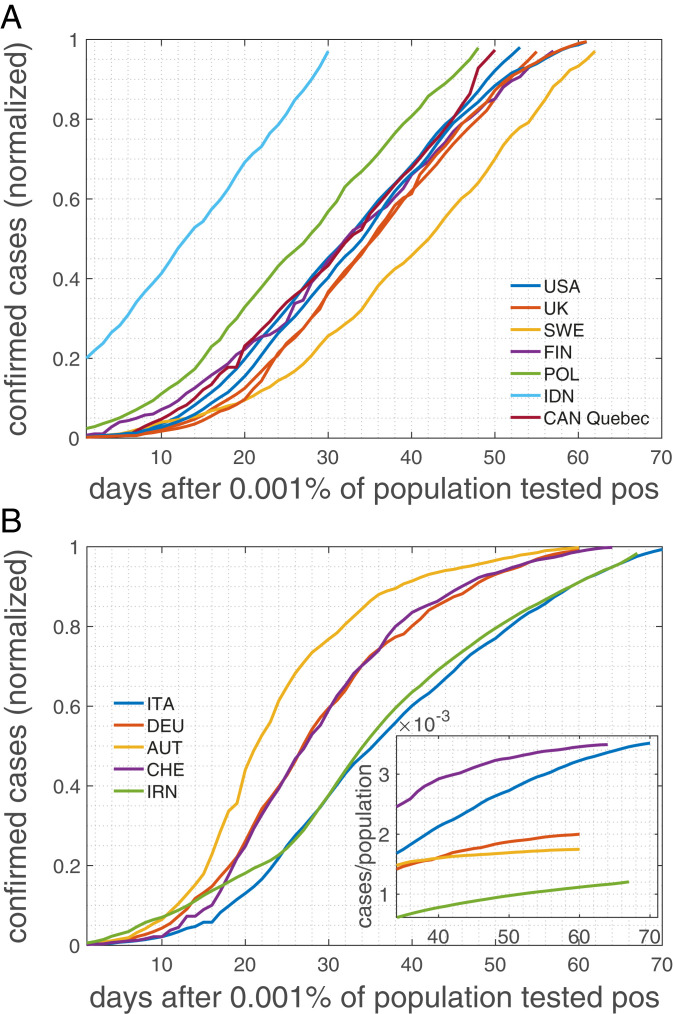
Cumulative numbers of positively tested cases normalized to the last day (8 May 2020). Countries, even though many followed radically different strategies in response to the pandemic, seem to belong to one of three groups: (*A*) countries with a remarkably extended linear increase of the cumulated number of positively tested cases, including the United States, the United Kingdom, and Sweden, and (*B*) countries with an extended linear increase that tends to level off and enter a regime with a smaller slope. *B*, *Inset* shows an extended regime after the peak (cases per population size).

Maybe the most striking observation in the COVID-19 infection curves is that they exhibit linear growth for an extended time interval quite in contrast to the S-shaped curves expected from epidemiological models. For a wide range of countries regardless of size, demographic and ethnic composition, or geolocation, this linear growth pattern is apparent even by a plain-eye inspection of the number of positive cases, e.g., ref. 13. In Fig. 1*A* we show infection curves (number of confirmed positive cases) for the United States, the United Kingdom, Sweden, Finland, Poland, Indonesia, and a province of Canada. Clearly, after a short initial exponential phase, infection curves are practically linear for several weeks. For many other examples, see ref. 13. Many countries that implemented NPIs in response to the COVID-19 crisis (12) show a different pattern. They also show an extended linear growth; however, infection curves tend to bend and level off in response to the implemented measures (Fig. 1*B*). The extent of the linear regime depends on the onset of the measures (12). Many countries that are still in the early phase of the pandemic (8 May 2020) show the initial almost exponential growth (*SI Appendix*, Fig. 1). According to basic epidemiological concepts, growth patterns with extended linear regions are not to be expected. They can be observed only if the infection growth rate equals the recovery rate, giving an effective reproduction number, R(t), of 1. Chances of observing such a behavior over an extended period in a country are extremely tiny, let alone in several countries. Mathematically speaking, linear growth is basically a measure zero solution in compartmental models.

The basic question of this paper is to clarify the mechanism that keeps R(t)∼1. In classical susceptible–infected–recovered (SIR) (9) models there are no terms that explicitly peg R(t) to 1 (*SI Appendix*). A simple explanation could be a limiting capacity of availability of test kits. If the daily number of tests is limited and assuming a fixed ratio of confirmed cases per test, linear growth in the number of positively tested would be the consequence. However, most European countries, even though experiencing initial difficulty with testing capacity, have, by now, enough tests available.

The rationale underlying social distancing efforts is that they lead to a reduction of contacts which essentially makes the social network sparser (12). Infections occur if 1) there is a social interaction between an infected and a susceptible person and 2) this contact is intense enough to lead to a disease transmission. For instance, given a basic reproduction number of $R_{0}∼3$, we effectively reach herd immunity if two of three contacts are avoided. Still, this does not yet explain linear growth as a slight increase or decrease in contact probabilities would again lead to a faster-than-linear growth or suppression, respectively. Network density alone cannot explain persistent linear growth.

In classic epidemiology network effects have long been ignored in favor of analytical tractability (14). In that case epidemiological models can be formulated as differential equations, assuming that every person in principle can infect any other. This is called the well-mixed or mean-field approximation (*SI Appendix*). However, that fact that networks matter in epidemiology has been recognized for almost two decades and has led to extremely relevant contributions, such as the dependence of vaccination thresholds on network topology (15). Classic contributions such as refs. 16 and 17 were able to incorporate network topology into analytically solvable SIR models. There it is possible to solve the SIR model in terms of outbreak size and epidemic size; however, no focus was put on the details of infection curves below the epidemic limit. When dealing with structured networks, it might well be that the mean-field approximation does no longer hold, and details of the networks start to become crucial.

Since social networks are key to understand details of epidemic outbreaks, what do they look like? The answer is highly nontrivial since social networks are hard to define. In terms of network topology, it became clear that they are neither pure random graphs, nor small-world networks, nor purely scale-free. They are of a more involved structure, including multilevel organization (18); weak ties between communities (19); and temporal aspects that suggest a degree of fluidity, however, with stable social cores (20).

Here we try to understand the origin of the extended linear regime in infection curves, as currently observed in the number of positively tested cases in the COVID-19 pandemic across many countries. To this end we solve the SIR model on a simple social network and report a hitherto unobserved transition from linear growth to S-shaped infection curves. We show that for a given transmission rate there exists a critical degree below which linear growth is expected and above which the model reproduces the classical SIR results. Below the critical degree the mean-field approximation starts to fail. For the underlying social networks we use a Poissonian small-world network that tries to capture several empirical facts, including a heterogenous number of social links (degree), the small-world aspect, the fact that people tend to live in small groups (families), that these groups overlap, and that work and leisure relations can link distant groups (*Methods*). The framework allows us to model a lockdown as a change in social networks with a high degree to one with a degree that characterizes the members of a household. Based on data on household size in the European Union (21), on empirical estimates on how long individuals are contagious, and on transmission (or attack) rates we are able to calibrate the model to real countries. In particular, we compare the situation in the United States and Austria. These countries differ remarkably in size and the measures taken in response to the COVID-19 pandemic (12). While Austria imposed a lockdown relatively early on in combination with a number of other measures, the United States has implemented measures hesitantly with the consequence that the situation was “not under control,” as Dr. A. Fauci, an advisor to the Trump administration, stated on 12 May 2020 (22). The model reproduces the real infection curves to a remarkable degree. All parameters are empirically motivated; there are no fitted parameters involved.

## Model Dynamics.

We assume that there are N individuals connected by social links. If i and j are connected, $A_{i,j}=1$; if they are not, $A_{i,j}=0$. As a toy model for social networks we use a so-called small-world network with average degree D and shortcut probability ϵ (*Methods*). The small-world aspect allows us to model transmission between local groups and “superspreaders” (23). As in a SIR model, every individual is in one of three possible states, susceptible (S), infected (I), and recovered (R). If an individual is infected, the individual will infect susceptible neighbors with a per-day transmission probability, r. This means that on every single day the probability of passing the infection to a susceptible neighbor is r, which is sometimes called the microscopic spreading rate (17). Once a person is infected that person stays infectious for d consecutive days. After this the person can no longer infect others and is called recovered. Once recovered the state will no longer change. The update rules of the corresponding model are as follows: Initialize all nodes as susceptible; select $N_{ini}$ nodes randomly and change their state to infected. At every timestep t, find all infected and infect their susceptible neighbors with probability r. Set all infected nodes that have been infected for more than d timesteps to recovered. And proceed to the next timestep until the dynamics come to a halt. All nodes are now either recovered or susceptible.

At every timestep (day), t, we count the number of new cases, C(t); the infection curve of positive cases, P(t), is the cumulative sum of C(t). The model parameters are related to those of the SIR model (*SI Appendix*) by γ=1/d, and β=rD/N. If the underlying network fulfills the conditions necessary for the mean-field approximation, C(t) corresponds to R(t) up to a timeshift of d.

## Results

### Infection Dynamics.

**Fig. 2.**
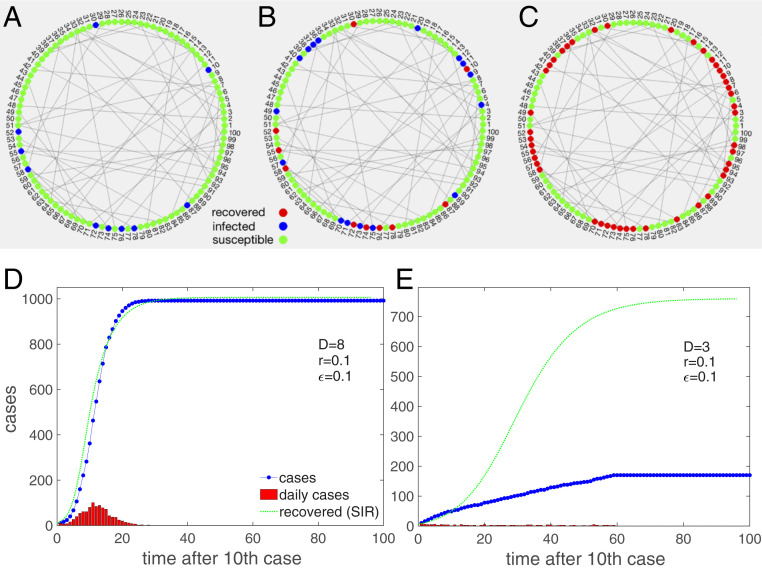
Schematic demonstration of the model. Nodes are connected in a Poissonian small-world network. Locally close neighbors resemble the family contacts, and long links to different regions represent contacts to others, such as people at work. (*A*) Initially, a subset of nodes is infected (blue), and most are susceptible (green). (*B*) At every timestep, infected nodes spread the disease to any of their neighbors with probability r. After d days infected nodes turn into “recovered” and no longer spread the disease. (*C*) The dynamics end when no more nodes can be infected and all are recovered. (*D*) Infection curve P(t) (blue dots) for the model on a dense Poissonian small-world network, D=8. The daily cases (red) first increase and then decrease. For comparison, we show the recovered cases, R(t), of the corresponding SIR model with γ=1/d, and β=rD/N (green). The mean-field conditions are obviously justified to a large extent. (*E*) Situation for the same parameters except for a lower average degree, D=3. The infection curve now increases almost linearly; daily increases are nearly constant for a long time. The dynamics reach a halt at about 17% infected. The discrepancy to the SIR model (green) is now obvious.

We demonstrate the model schematically in Fig. 2 *A*–*C*. In the limit of large degree D and large ϵ the model should approximately fulfill the mean-field conditions and should be close to a classical SIR model. This is seen in Fig. 2*D* where the trajectory of an infection curve, P(t), is shown (blue dots) for a network of 1,000 nodes with a degree of D=8, ϵ=0.1, a period of contagiousness of d=6 d, and a transmission rate of r=0.1; 10 nodes were infected at the start. The situation closely resembles the solution of the recovered, R(t), the SIR model with γ=1/d, and β=rD/N, shown as the dotted green line. Note that a timeshift of −d days is necessary to compare P(t) and R(t). The daily cases (red) increase, reach a peak, and decrease. The typical exponential initial phase in P(t) is seen, immediately followed by a quick relaxation of growth until the plateau forms at the herd immunity level (in this example at 98%).

The infection curve, P(t), changes radically when the degree of the network is lowered to D=3 (all other parameters kept the same) (Fig. 2*E*). Clearly, it increases almost linearly for a remarkable timespan, which is in marked contrast to the SIR expectation (green line). The situation already resembles the situation of many countries. Once the system converged to its final state, only about 17% of nodes were infected, which is far from the expected (SIR) herd immunity level of about 77%.

The change of the infection curve from the S-shaped to a linear behavior is clearly a network effect and indicates that the mean-field assumptions might be violated. To understand this better we next study the parameter dependence more systematically.

### Parameter Dependence and Phase Transition.

**Fig. 3.**
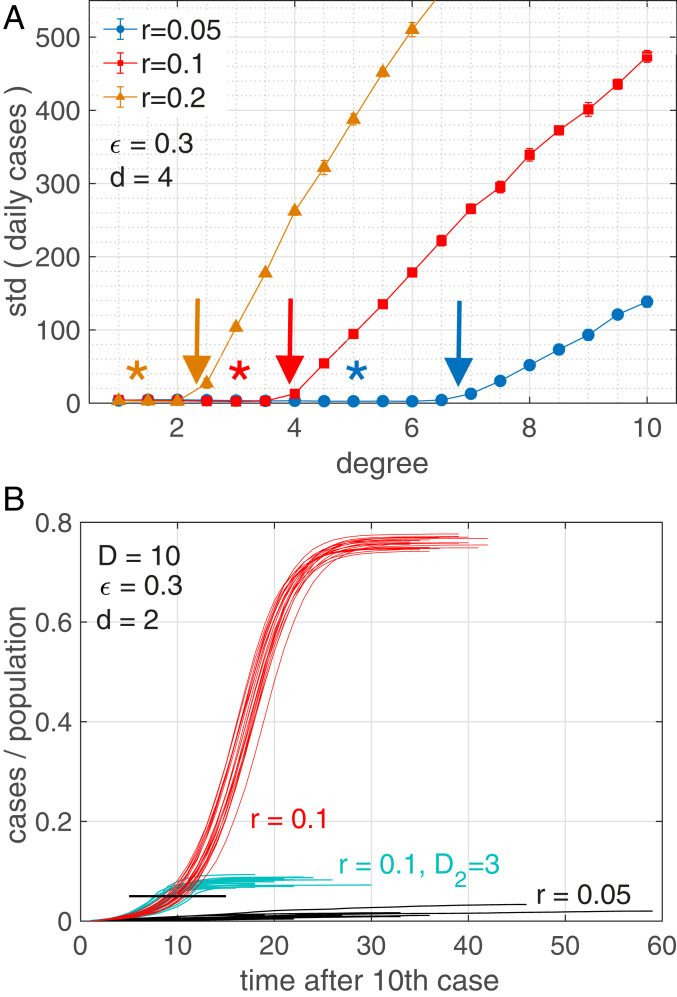
(*A*) Order parameter for the transition from linear to S-shaped infection curves as a function of degree, D, for transmission rates r=0.05 (blue), 0.1 (red), and 0.2 (orange). The transition happens at the critical points, $D_{c}$, where the order parameter starts to diverge (arrows) (Table 1). The asterisks in Fig. 3*A* denote the degree, $D_{sir}$, at which the SIR model would show a linear curve, $D_{sir}=1/dr$. Colors correspond to the respective r. (*B*) Infection curves (20 realizations) for three scenarios for a network with D=10. Red scenario: At a transmission rate of r=0.1 we see S-shaped curves reaching herd immunity at about 75%. Black scenario: For the same network with a lower transmission rate, r=0.05, we fall below the critical degree $D_{c}$ and consequently observe linear growth; note the convergence of infected at levels of 1 to 4%, which are very much below herd immunity (75%). Turquoise scenario (lockdown): We start with the same network with r=0.1, as in the red scenario. After 5% of the population (black bar) is infected there is a lockdown that changes the network to one of degree $D_{2}=3$, from one day to the next. The S-shaped growth immediately stops and levels off at about 10% infected. Other parameters: d=2, ϵ=0.3, and N=10,000; 10 initially infected.

We are interested to see whether there is a critical degree, $D_{c}$, below which the infection curve is (quasi)linear, whereas for $D>D_{c}$ it assumes the S shape. For this we define an appropriate “order parameter,” O, able to distinguish linear from S-shaped growth, namely the SD of daily increments of infected people (*Methods*). In Fig. 3*A* we show this order parameter as a function of the degree, D, of the network for three transmission rates r=0.05, 0.1, and 0.2 (obtained as averages over 10 independent realizations with randomly selected 10 initially infected). It is clear that at specific (critical) degrees, $D_{c}$, the order parameter switches from (close to) zero to larger values. The position of the critical degrees depends on the parameter settings (Fig. 3*A*, arrows). It decreases with the transmission rate r; while for r=0.05 we find $D_{c}=6.6$, for r=0.1 it is $D_{c}=3.8$, and for r=0.2, we have $D_{c}=2.3$. The critical degree also decreases with the parameters ϵ and r. For more parameter settings, see Table 1 and *SI Appendix*, Fig. 2. The asterisks in Fig. 3*A* denote the degree, $D_{sir}$, at which the SIR model would show a linear curve, $D_{sir}=1/dr$. Colors correspond to the respective r.

**Table 1.** Critical degree, $D_{c}$, for a range of parameter values for the transmission rate, r, rewiring probability, ϵ, and duration of infectiousness, d

	d	r = 0.015	r=0.05	r=0.1	r=0.2
ϵ=0.1	d=2		12.5 (19.2)	7.4 (10.1)	4.3 (5.5)
	d=4		7.9 (10.1)	4.8 (5.5)	3.0 (3.3)
	d=6		5.7 (7.1)	3.5 (4.0)	2.4 (2.5)
ϵ=0.3	d=2		12.2 (16.4)	6.3 (8.7)	3.3 (4.8)
	d=4		6.6 (8.7)	3.8 (4.8)	2.3 (3.0)
	d=6		4.7 (6.1)	2.9 (3.6)	1.9 (2.3)
	d=14	7.2 (8.3)	2.7 (3.2)	1.9 (2.1)	1.5 (1.5)
ϵ=0.5	d=2		11.7 (14.3)	5.9 (7.7)	3.2 (4.3)
	d=4		6.0 (7.7)	3.3 (4.3)	2.2 (2.7)
	d=6		4.4 (5.4)	2.7 (3.2)	1.8 (2.1)

*N* = 10, 000. Numbers in parentheses refer to the theoretical results from Eq. **1**.

We checked that the position of the critical degrees is relatively robust under the size of the network and variations in topology. We find that for N=1,000 and 10 initially infected, the critical degrees are practically at the same locations. Regarding the topology, we implemented a standard small-world network with a fixed degree. Also here, results are practically identical (*SI Appendix*, Fig. 3).

For the Poissonian small-world network we are able to estimate the critical degree analytically by a “fuse model”

$$D_{c}∼1+2/\left(rd\left(1+\epsilon \right)\right).$$ [1]

For the derivation, see *SI Appendix*, Text S6. This result slightly overestimates the simulation results (Table 1). However, for large values of r and ϵ, theoretical predictions and simulation results are in remarkable agreement. Indeed, the used second-order approximation systematically underestimates the spreading velocity, i.e., overestimates the number of infected in active regions of the network, which in turn leads to an overestimation of $D_{c}$ (*SI Appendix*, Text S6). Also finite size effects in the simulation may add to the observed deviations.

The existence of critical degrees signals the presence of a hitherto overlooked transition between linear and S-shaped growth that is most likely due to the fact that the well-mixed or mean-field assumption breaks down below $D_{c}$. To illustrate the dependence of this transition on the transmission rate, Fig. 3*B* shows 20 realizations of model infection curves for a network with D=10 at a rate of r=0.1 (red). The curves were obtained for 20 different initial conditions in the choice of the 10 initially infected nodes. One observes typical S-shaped curves reaching herd immunity at about 75%. Note that R(t→∞) of the SIR model reaches about 80%. For the same network with a lower transmission rate of r=0.05, which is well below the critical degree, we are in the linear growth domain (turquoise). The maximum of infected reaches levels of only 1 to 4%, which are drastically lower than SIR herd immunity with R(t→∞)∼ 15%. The 20 black infection curves depict a “lockdown” scenario: We start with the same network with r=0.1 (red). On the day when 5% of the population is infected (black bar) a lockdown is imposed which means that effectively the social network changes from one day to the next. We model this by switching to a Poissonian small-world network with a low degree, $D_{2}=3$. All other parameters are kept identical. S-shaped growth stops and final infection levels of about 10% are obtained.

We confirm that the mechanism to obtain linear infection curves is present also for more realistic social contact networks (20, 24, 25), by running the algorithm on networks derived from actual contact networks that are publicly available (26). For the situation where the degree of these networks falls below $D_{c}$, we typically observe linear infection curves. Details are presented in *SI Appendix*, Fig. 7.

### Calibration.

We calibrate the model to the COVID-19 infection curves of two countries, the United States and Austria, to demonstrate its potential applicability for estimating the effects of NPIs. For this we have to make the following assumptions on the model parameters:

The viral dynamics of COVID-19 are highly heterogenous (27). Motivated by evidence that people carry viral loads and thus can be contagious for more than 20 d after disease onset (most people are contagious for shorter periods) (28, 29) and given that infectiousness can start 2 to 3 d before showing symptoms (28), we use d=14 d.

In 2019 the average household size in the European Union was 2.3 people (21). If we assume that at work and during leisure activity on average one meets 3 to 4 people more per day, we decide to use an average degree of D=5 in our Poissonian small world for normal conditions. If we assume that on average about 30% of all of our social relations are outside of our household, we set ϵ=0.3. This is a somewhat arbitrary choice; however, note that deep in the linear regime, ϵ is found to be an almost irrelevant parameter that does not influence the outcome in significant ways. To model the lockdown that was imposed in Austria on 16 March 2020 as an NPI we assume that its effect is basically to reduce social contacts to within households and eliminate any other contacts. For this scenario we assume D=2.5 and ϵ=0. Finally, for the daily transmission rate we set r=0.0149. This choice is motivated by estimates of the COVID-19 individual-level secondary attack rate (SAR) in the household setting, which is reported at about 19% (30), and the relation $r=1-{\left(1-SAR\right)}^{1/d}$. Note that these estimates of the parameters are based on recent estimates (not yet peer reviewed) and might change in the future. Also note the SIR limit for this case is at R(t→∞)∼ 20%, which is somewhat lower than what is expected in refs. 7 and 8. With these parameters we find a critical degree of $D_{c}=7.2$. For a detailed discussion of the model parameters in the context of NPIs and how they change under different strategies, see *SI Appendix*, Text S7.

We use 100,000 nodes and 40 and 100 initially infected for the United States and Austria, respectively. Since it is not possible to compute every individual in the simulation, we decided to initiate the simulation at the point where 0.1% of the population tested positive, that is, 7 April for the United States and 3 April for Austria. For the respective population sizes we use United Nations data from 2019 (31).

**Fig. 4.**
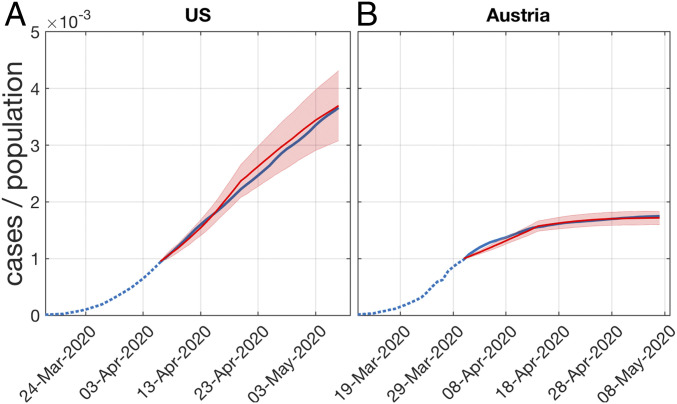
Model infection curves (red) when calibrated to the COVID-19 curves of positively tested in (*A*) the United States and (*B*) Austria. Five realizations with different sets of initially infected are shown. The simulation starts when more than 0.1% of the population tested positive. The situation in the United States assumes a Poissonian small-world network with average (daily) degree D=5. The lockdown scenario in Austria that has been in place from 16 March to 15 May 2020 is modeled with social contacts limited to households, D=2.5. For the choice of the other model parameters, see main text. The model clearly produces the correct type of infection curves.

In Fig. 4 we show the model infection curves in comparison to the number of positively tested persons (13) for the United States (Fig. 4*A*) and Austria (Fig. 4*B*). Solid blue lines mark the situation where more than 0.1% of the population tested positive; simulations are performed from that date on. Note that one case in the model represents many in reality. In the simulations relatively few cases are produced and the integer steps are still visible. Obviously, the model produces infection curves of the observed type.

We discuss the role of superspreaders in two additional simulations, one where we introduce superspreaders defined as individuals with a much higher degree than the population average and the other where superspreading comes from individuals which are much more contagious but have similar degrees to the rest of the population. Results are presented and discussed in *SI Appendix*, Fig. 4. Both types of superspreaders do shift the critical degree toward lower levels, as expected.

Finally, we performed a cross-correlation analysis of the actually measured $R_{e\mathrm{ff}}$ of Austria versus the fluctuations in mobility reduction that we can obtain from mobile phone data of individual Austrian states. We present the cross-correlations in *SI Appendix*, Figs. 9 and 10. We can exclude the a priori possibility (32) that mobility fluctuations influence $R_{e\mathrm{ff}}$ strongly enough to explain why it fluctuates around 1. For details, see *SI Appendix*, Text S9.

## Discussion and Conclusions

Here we offer an understanding of the origin of the extended linear region of the infection curves that is observed in most countries in the current COVID-19 crisis. This growth pattern is unexpected from mainstream epidemiological understanding. It can be understood as a consequence of the structure of low-degree contact networks and appears naturally as a hitherto unobserved (phase) transition from a linear growth regime to the expected S-shaped curves.

We showed that for any given transmission rate there exists a critical degree of contact networks below which linear infection curves must occur and above which the classical S-shaped curves appear that are known from epidemiological models. The model proposed here is based on a simple toy contact network that mimics features of a heterogenous degree, the small-world property, the fact that people tend to live in small groups that overlap, and the fact that distant groups are linked through work and leisure activities. We showed how the model can be used to simulate the effects of NPIs in response to the crisis by simply switching to low-degree networks that do not allow for linking of distant groups.

The model not only allows us to understand the emergence of the linear growth regime, but also explains why the epidemic halts much below the levels of herd immunity (given no in-flow of infected). Further, it allows us to explain the fact that in countries which are beyond the (first) maximum of the epidemic, a relatively small number of daily cases persist for a long time. This is because small alterations and rearrangements in the contact networks will allow for a very limited spread of infections.

We find that for the empirically motivated parameters used here, the critical degree is $D_{c}=7.2$, which is above the degree of the contact networks for which we effectively assume D∼5. This means that linear growth must be expected. Note that countries with larger family structures might be closer to the critical degree, above which catastrophic epidemic spreading would occur.

The linear growth phase appears to be dominated by cluster transmission of the disease, meaning that new infections primarily appear in the “small-worlds” or local network neighborhood (households, workplaces, etc.) of infected individuals. In the superlinear (exponential) phase, sustained community transmission sets in where new cases cannot be traced to already known cases in their neighborhood. In this regime, transmission across the shortcuts in the network becomes more prevalent. This effective mixing of the population gives a dynamic that approaches the mean-field case of SIR-like models.

Finally, we calibrated the model to realistic network parameters, transmission rates, and the time of being contagious and showed that realistic infection curves (examples of the United States and Austria are shown) emerge without any fine-tuning of parameters. The onset of the NPI (lockdown)—and the associated reduction of the degree in the contact networks—determines the final size of the outbreak which is well below the levels of herd immunity. We demonstrate the importance of timing of the interventions in *SI Appendix*, Fig. 6. In *SI Appendix*, Text S7 we discuss the impact on the Austrian infection curve if the same NPI would have been implemented 10 d later (*SI Appendix*, Fig. 6*B*). An increase of about 30% of cases is observed. In the same spirit, in *SI Appendix*, Fig. 6*A* we demonstrate what could have happened if NPIs, with similar effects to those that were implemented in Austria, had been installed in the United States. The results indicate that about half of the cases could have been avoided (at the beginning of May 2020).

For a more detailed discussion of the applicability of the presented model in terms of modeling the effects of NPIs, refer to *SI Appendix*, Text S7. In *SI Appendix*, Text S8 we discuss three additional case studies of the infection curves of China (Hubei), Singapore, and South Korea. We implement temporal sequences of changes in the model parameters that roughly resemble the effects of the NPIs implemented in reality. We recover the basic features of the actual infection curves to a remarkable extent, at least qualitatively (*SI Appendix*, Fig. 8).

The two types of superspreading, the network based and transmission based, both lead to a clear finding that the presence of superspreaders shifts the critical degree toward lower levels. However, the presented mechanism to obtain linear infection curves remains fully intact. The message for Austria and the United States is that in both countries the density of superspreaders is not high enough in the considered observation period to shift the critical degree toward exponential growth, given the effective degrees in the populations.

Given the number of countries that entered linear growth phases, our results raise serious concerns regarding the applicability of standard compartmental models to describe the containment phase achieved by means of NPIs. SIR-like models show linear growth only after fine-tuning parameters and linear growth would be a mere statistical fluke. We argue that network effects must be taken into account to understand postintervention epidemic dynamics.

## Methods

### Poissonian Small-World Network.

For the network A we use a Poissonian small-world network, which generalizes the usual regular small-world network in the sense that the degree is not fixed, but is chosen from a Poissonian distribution, characterized by λ. The network is created by first imposing a Poissonian degree sequence on all nodes. Assume that nodes are arranged on a circle. Nodes are then linked to their closest neighboring nodes on the circle. This creates a situation where every person is a member of a small local community. As for real families, these communities strongly overlap. Finally, as for the conventional small-world network, with probability ϵ we relink the links of every node i to a new, randomly chosen target node j, which can be far away in terms of distance on the circle. ϵ is the fraction of an individual’s social contacts that are outside the local community (family). These links can be seen as links to colleagues at work or leisure activities and allow us to model the existence of superspreaders (23). Note that the actual average degree of the so-generated network is very close to the λ of the Poisson distribution, D∼λ. We also implemented a conventional small-world network with a fixed degree. When results are compared with the Poissonian small-world network, only marginal differences are observed.

### Order Parameter.

To distinguish the linear from the sigmoidal growth, we propose a simple “order parameter” as the standard deviation (SD) of all new daily cases (after excluding all days where there are no new cases),

O=SD(C(t)). [2]

Clearly, for a linear growth of the infection curve, daily cases, C(t), are constant, and the SD is zero. For the S-shaped growth, daily cases first increase and then decrease over time, and the SD becomes larger than zero. Hence, a SD deviating from zero signals the presence of a nonlinear increase of the cumulative positive cases, P(t).

## Supplementary Material

Supplementary File

## Data Availability

All study data are included in this article and *SI Appendix*.
