# Supplementary material for: A network-based explanation of why most COVID-19 infection curves are linear
Source: Proc Natl Acad Sci U S A. 2020 Aug 24;117(37):22684–9. doi: 10.1073/pnas.2010398117 (PMC7502715; doi:10.1073/pnas.2010398117)
Supplement: Supplementary File [file pnas.2010398117.sapp.pdf]

Supporting Information

Text S1: The SIR model

The classic epidemiological model is the SIR model that models the time evolution of compartments of susceptible S(t), infected I(t), and recovered R(t) individuals. It can be formulated as the set of non-linear differential equations,

$$\begin{aligned} \frac{d}{dt}S &= -\beta\frac{SI}{N} \\ \frac{d}{dt}I &= \beta\frac{SI}{N} - \gamma I \quad . \\ \frac{d}{dt}R &= \gamma I \end{aligned} \tag{1}$$

At any point in time, $S+I+R = N$. To solve the equations, for a given N the initial conditions, $S(0)$ and $I(0)$ must be specified. γ is called the recovery rate, β is the infection rate, controlling how often a susceptible–infected contact results in a new infection. To play with it online, see e.g. `http://www.public.asu.edu/~hnesse/classes/sir.html?Alpha=0.3&Beta=0.1666&initialS=990&initialI=10&initialR=0&iters=70`

Text S2: Countries in early phase of the pandemic

In SI Fig. 1 we show several countries that display the typical initial exponential growth of the infection curve. Many of these countries are in South America.

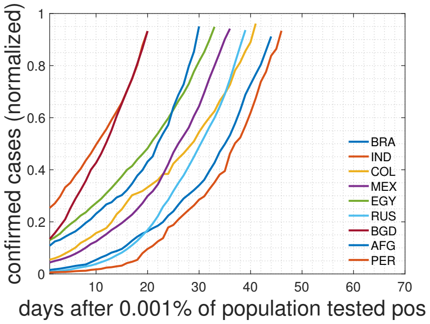

Figure 1: Cumulative numbers of positively tested cases, as in Fig. 1 (main text), normalized to the last day (May 8, 2020). for countries with an exponential growth, possibly because they are still in an early phase.

www.pnas.org/cgi/doi/10.1073/pnas.2010398117

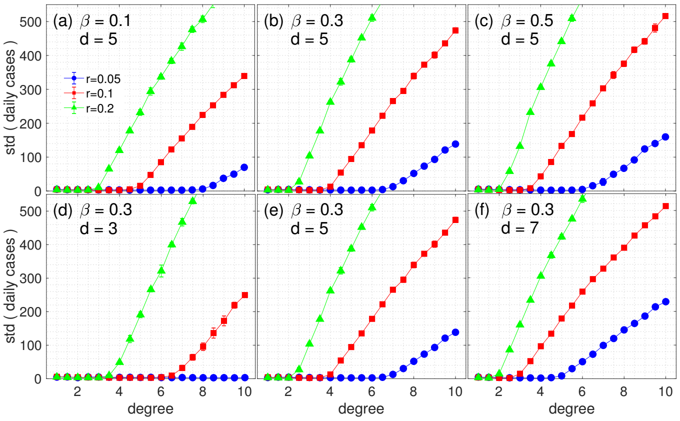

Figure 2: Transitions from linear growth ($\mathcal{O} \sim 0$) based on a Poissonian small-world networks with $N = 10,000$ with degrees ranging from 0 to 10. Cases for transmission probabilities of $r = 0.05$, 0.1, 0.2, and for rewiring probabilities $\epsilon = 0.1$, 0.3, and 0.5 are shown. Days of being contagious are $d = 2$, $d = 4$, and $d = 6$.

Text S3: Position of critical degrees for various parameter settings

In SI Fig. 2 we see the position of the transition from linear to traditional epidemic growth in terms of critical degrees. The order parameter O is plotted on the y-axis. In traditional sigmoidal growth O takes values well above zero, the onset defines the critical degree,D_c, for the various parameter settings, collected in Table 1. The position of critical degrees is practically the same with a network of size $N = 1000$, assumed that both are initialized with 10 initially infected.

Text S4: Critical degrees for various parameter settings on a small-world network

The positions of the critical degrees (order parameter becoming larger than 0) are shown for a standard small-world networks with $N = 1000$ in SI Fig. 3. Note that here we have used an even degree, indicated by $2K$.

Text S5: Super-spreaders and super-infectors

One of its important features in the COVID-19 pandemic is the existence of active asymptomatic carriers of the virus. They may cause a significant contribution to the viral spread. They can be detected by massive random screenings

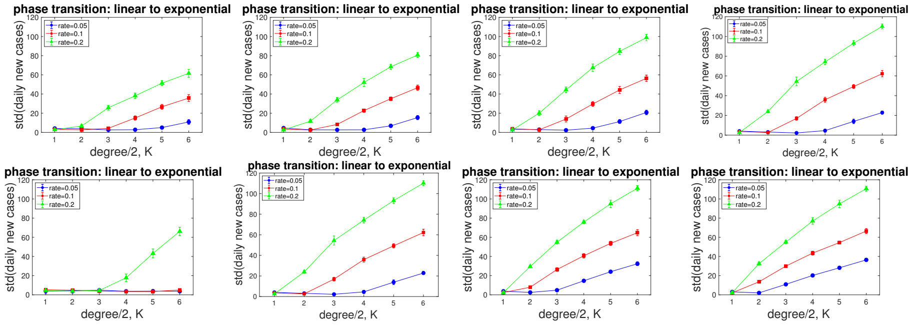

Figure 3: Position of critical degrees for a standard small-world networks with $N = 1000$, with degree $D = 2K$ and transmission rates of $r = 0.05$, 0.1, 0.2. (top) Rewiring parameters shown are $\epsilon = 0.05, 0.1, 0.2, 0.3$. for $d = 5$ days being infectious. (bottom) Here we set $\epsilon = 0.3$ and vary the days being contagious: $d = 1$, $d = 4$, $d = 6$, $d = 9$. In all cases 10 randomly chosen nodes were initially infected.

or by identifying contact persons. Also characteristic for the spread of COVID-19 are so-called super-spreaders, where it has been estimated that 80% of all infections have been caused by 10% of infected cases [1].

To discuss the role of super-spreaders in the presented framework, we distinguish between two types. The first is a super-spreader because it has a much higher number of connections than an average individual (e.g., barkeepers in Tyrolean pubs, singers in choirs, instructors in fitness classes, etc.). The other type of super-spreader could be people that are more infectious than others. We study the effects of both types in two different simulations. Results for the critical degree, D_c, are shown in SI Fig. 4.

Network Super-spreaders

We use the same Poissonian small-world network type as in the main text with the same parameters as those shown in Fig. 3 (a) in the main text ($r = 0.1$, $\epsilon = 0.3$, $N = 10000$, 10 initially infected). The degree of the nodes is shown on the x axis. We assign $N_{ss} = 50$ nodes (0.5 % of the population) to be super-spreaders that are endowed with a fixed degree of 20. These links are established to randomly chosen nodes in the network. The red curve shows the order parameter for the case with super-spreaders with respect to the reference case (blue) that is the same curve shown in Fig. 3 (a). Obviously, the critical degree shifts from about 3.8 (blue) to about 3.0 (red). This is to be expected, since the network with super-speaders has a higher number of links. If we randomly prune the network, so that the total number of links is the same as in the original Poissonian small-world network, we obtain the green line. There the effect is smaller, but now directly comparable (in terms of average degree)

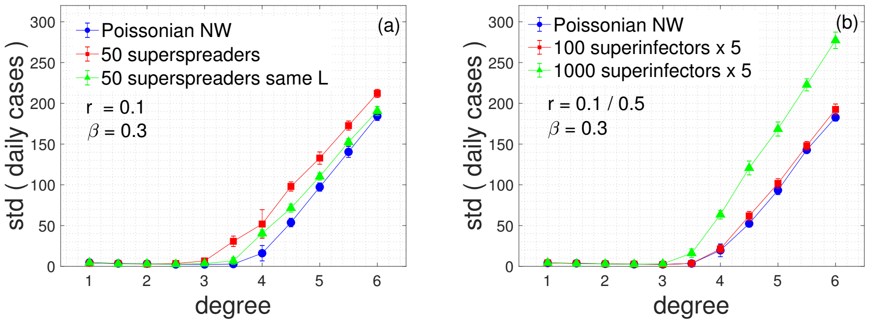

Figure 4: Order parameter as a function of the degree. The blue curves in both panels is the same line as the blue line in Fig. 3 (a) in the main text. It serves as the reference line to compare to the shift of the critical degree in the case of super-spreaders (a) that have a much higher degree (x-axis). For the presence of 50 super-spreaders (0.5% of the population) with a degree of 20 (red curve) there is a clear shift of the critical degree to the left, from about 3.8 (blue) to about 3.0 (red). The green line represents the case of 50 super-spreaders (0.5% of the population) with a degree of 20, however with a random removal of 1000 links from the network, so that the blue and green curves have the same number of links. (b) Case for super-infectors. A fraction of 1% super-infectors that are 5 times more infectious than others (red) leaves the results unaltered. Only when about 10% are highly infectious the shift in D_c becomes significant. ($r = 0.1$, $\epsilon = 0.3$, $N = 10,000$, 10 initially infected).

with the blue case. There the critical degree is about 3.6. We also simulated a higher degree of super-spreaders of 50, which shifts the critical degree further to the left.

Super-infectors

The other possibility of super-spreading infected is that they have a drastically higher probability to infect others. In two simulations, we randomly select $N_{ss} = 100$ (red) and 1000 (green) nodes who are five times more infectious ($r = 0.5$) than the rest of the population ($r = 0.1$). The reference curve is again the same blue curve as in Fig. 3 (a). We see that a 1% fraction of super-infectors in the population has practically no effect on the the critical degree (red curve sits on top of blue one within errors), however, when the fraction is raised to 10% the D_c is shifted from about 3.8 (blue) to about 3.4 (green).

We conclude that the role of super-spreaders of both types does indeed have an effect on the location of the critical degree. D_c appears to be highly sensitive to those super-spreaders that are characterized by a high degree in their network,

while the infectiousness appears to be less relevant. An very high fraction of about 10% super-infectors is needed to shift D_c from about 3.8 (blue) to about 3.4 (green). This finding again supports the intuition that it is the network that forces R towards 1. The message for the countries of Austria and the US is clear. In both countries the density of super-spreaders is not high enough to shift critical degree towards a level, where exponential growth would be observed.

Text S6: Analytical derivation of the critical degree in terms of model parameters

In statistical physics it is known that the dimensionality of a system is crucial in whether a mean-field approach is suitable for understanding the properties of a system. For the Ising model only systems with a dimension 4, and larger, will be described sufficiently well by a mean-field model. Similarly, on networks with non-regular network topologies, the average degree (if the network is not too heterogenous) will decide whether the mean-field solution will be sensible.

In the paper we use ring-shaped Poissonian small-world networks with average degree, D, and rewiring probability ϵ. For simplicity we consider this contact network to consist of a (almost) regular network with on average $D(1-\epsilon)$ short-range links (family, spouses, or caregivers, etc.) per node and $D\epsilon$ links that are are shortcuts connecting overlapping local groups. r is the (average) probability that a risky interaction with an infected, in one day, leads to an infection in the susceptible; d is the (average) number of days a person can infect another. The probability that one infected person will infect a susceptible person under repeated contact over d days is $r_d \equiv 1 - (1-r)^d \;\sim\; rd$.

Following textbook knowledge, the multiplication factor R is $R = Dr_d$, and the critical connectivity D_c of a person is given by the condition $R = 1$, i.e. $D_c = 1/rd$. If $D > D_c$, then $R > 1$ the infection spreads exponentially, i.e. the number of daily cases is $C(t) \propto R^{t/d}$, where t is the time to the initial infection. For $D < D_c$ the infection dies out exponentially and the cumulative number of infected $P(t) = \sum_t C(t)$ is small with respect to the size of the population N. For $D = D_c$, the number of infected will behave critically, i.e. $P(t) \propto t^\alpha$, where α depends on the topology of the contact network, which for *simply connected* short-range contact networks, embedded in a two-dimensional world, could range between $\alpha \sim 1$ and $\alpha \sim 2$.

In other words, if the network topology resembles a regular two dimensional grid, we have $\alpha \sim 2$, because the number of infected will be proportional to the area, a radially spreading-infection front will cover. That is the number of newly infected is proportional to the length (circumference) of the (circular) infection front. If, however, the effective contact network topology is organized as a tree and a collection of essentially one-dimensional chains, the infection front spreads linearly like a flame along a fuse, and $\alpha \sim 1$.

Which power $1 < \alpha < 2$ is actually realized in a particular country or region again depends on the effective networks, which can be strongly influenced and shaped by non-pharmaceutical interventions (NPIs).

We now try to take dimensional effects into account in our derivation the critical degree, D_c. We can also explicitly consider the curvature, $\kappa = \pm 1/\mathcal{R}_{\rm front}$, of the infection front, where $\mathcal{R}_{\rm front}$ is the radius of the circle that can be fitted into an infection front. The sign of κ is positive if the infection spreads in a circular shape (concave case), it is negative if the infections surround an island of susceptible population (convex case). For the model presented in the main text, the infection front is flat, $\kappa = 0$.

Note that the expression $R = Dr_d$ is *exact* only for the first infectious person. Some that get infected later find at least one infected person already among their contacts, which modifies $R = Drd$ to

$$R = (D - n)rd\,, \tag{2}$$

where n is the effective number of persons among my interactions who I can no longer infect, either because, they are infected or recovered. $\nu = 1 - n/D$ is the fraction of susceptible. If we can determine n, then the condition $R = 1$ determines the critical connectivity

$$D_c = n + \frac{1}{rd}\,. \tag{3}$$

We now discuss two network types. The Erdős-Renyi (ER) random graph and the tree- or chain-like"fuse model". For low infection levels, one would intuitively guess that for the random graph $n \sim 1$ and for the "fuse-model", $n \sim (1 - \nu)D$, with $\nu \sim 1/2$, i.e. the "left hand" contacts of a newly infected person, already have been infected or have recovered, while the "right hand" contacts are still susceptible.

The case of the ER random graph

Let us briefly analyse the situation for a ER random graph with linking probability p, implying an average connectivity $D = Np$. Suppose the fraction of the susceptible population is ν, then the number of nodes that one infected node can *not* infect is $n = 1 + (D - 1)(1 - \nu)$. Inserting this into Eq. (3) yields

$$D_c = 1 + \frac{1}{\nu rd}\,. \tag{4}$$

For low infection levels, $\nu \sim 1$, we recover the naive intuition that $n \sim 1$. However, more importantly, one sees that D_c increases as the fraction of the susceptible population drops. Conversely, for a given connectivity D there exists a critical level

$$\nu_c = \min\left(1, \frac{1}{(D - 1)rd}\right)\,. \tag{5}$$

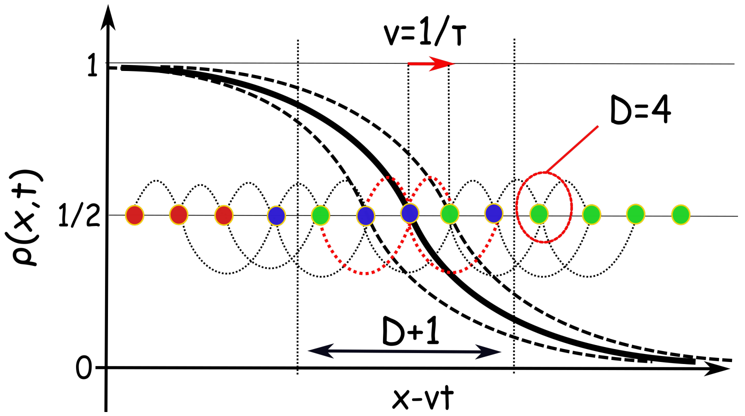

Figure 5: Propagation of the infection front along a regular network with connectivity $D = 4$. The front is approximated probabilistically by an invariant distribution function $\rho(x,t)$ that moves with a velocity $v = 1/\tau$ along the linearly organized network along the direction x. Red nodes are recovered or dead, green are susceptible, and blue are infected.

for the susceptible population. If $\nu \sim \nu_c$ the connectivity D becomes critical, and no exponential growth can appear. For the infection to die out quickly requires $\nu_c = 1$, i.e. $(D-1)rd < 1$, which further implies $D < 1 + 1/rd$. This is only possible if D has been sufficiently small at the time the initial infections happened.

The fuse model

Let us now look at a one-dimensional regular grid with each node having D contacts in its neighbourhood, as the model discussed in the main text. To this end, we introduce an invariant probability function, $\rho(x,t) = f(x - vt)$, that captures the dynamics of the infection front. It is depicted for the fuse model in Fig. (5). $\rho(x,t)$ is the probability that a node in location x is infected at time t. Nodes are connected with $D = 4$ others in a regular way, as seen. Red nodes are recovered (or dead), blue are infected, and green are susceptible. The black line is the "probabilistic infection front", $\rho(x,t)$.

It travels along the regular network of contacts at a constant velocity v along the x axis of the regular network. x measures the distance of the node from the initial infection in terms of intermediate nodes, t is the time passed since the initial infection in days. To estimate the velocity and the local density of susceptible persons at the infection front (which typically will differ strongly from the average level of susceptible persons in the population), we consider the

following equation

$$\rho(x,t+1) = \rho(x,t) + (1-\nu)2Dr\bar{\rho}(x,t)(1-\rho(x,t))\,, \tag{6}$$

that describes the probabilistic evolution of the probability of x to be non-susceptible at time $t+1$. The node either was non-susceptible at time t already, or x is susceptible and gets infected with a probability that is proportional to the number of non-susceptible nodes, assuming that the infection spreads with a velocity so that the non-susceptible nodes in a neighbourhood are in fact infected nodes. $\bar{\rho}(x,t)$ denotes a local average of ρ over the adjacent nodes. For a flat infection front, $\nu = 1/2$. Note that on a two-dimensional grid the radius of the infection front is given by $x = vt$ and at time $t+\tau$, the ratio of nodes that may have been infected to the number of nodes that may become infected can be approximated by $1 + \tau/t = 1 + v\tau\kappa$. Moreover, if we define τ as the time it takes $\rho(x,t)$ to travel one node along the line, then $\tau v = 1$, and $\nu = 1/(2+\kappa)$. If the infection front is flat, i.e. $\kappa = 0$, then as we expected naively before, we get $\nu = 1/2$. To be clear, ν does not measure the fraction of the entire susceptible population as it does in the ER random graph, but the fraction of the susceptible population in the neighbourhood (!) of the infection front only, where this fraction may be in fact close to $\nu \sim 1/2$.

If we approximate $\bar{\rho}(x,t) \sim \rho(x,t)$, then with the invariance condition

$$\frac{d\rho}{dt} + v\frac{d\rho}{dx} = 0\,, \tag{7}$$

one obtains the estimate

$$\rho(x,t) = \frac{1}{1+e^{\beta\tau x - t}}\,, \tag{8}$$

with $\beta = 2(1-\nu)Dr$. For this particular solution we have that $\rho(x,vt) = \rho(0,0) = 1/2$ marks the infection front. We next estimate the average probability, $\hat{\rho}$, that nodes to the left of the infection front are infected,

$$\begin{array}{rcl} \hat{\rho}(\tau) & \sim & \frac{1}{\nu D}\int_{-\nu D}^{0} dx\rho(x,0) \\ & = & 1 + \frac{1}{\tau\beta\nu D}\log\left(\frac{1+e^{-\tau\beta\nu D}}{2}\right) \\ & \sim & \frac{1}{2} + \frac{1}{8}\beta\nu D\tau + \mathcal{O}(\tau^3)\,, \end{array} \tag{9}$$

With this we can estimate the probability, q, that νD nodes infect the next node within a timespan, τ. We get

$$\begin{array}{rcl} q & \equiv & \sum_{m=1}^{\nu D}\binom{\nu N}{m}\hat{\rho}^n(1-\hat{\rho})^{\nu D - m}(1-r)^{(\tau-1)n}(1-(1-r)^n) \\ & = & \left(1-\rho\left(1-(1-r)^{\tau-1}\right)\right)^{\nu D} - \left(1-\rho\left(1-(1-r)^{\tau}\right)\right)^{\nu D} \\ & \sim & 2\nu\beta\hat{\rho}(1-r)^{\tau-1}\,. \end{array} \tag{10}$$

We obtain an equation in τ by asking how long it takes to increase the probability to be infected, $\rho(1,0) = 1/(1+\exp(\beta\tau))$, to the probability $\rho(0,0) = 1/2$. This equation reads

$$q\left(1 - \frac{1}{1+e^{\beta\tau}}\right) + \frac{1}{1+e^{\beta\tau}} = \frac{1}{2}\,. \tag{11}$$

It can be solved approximately with respect to τ, by expanding to second order in τ. Now that we have an estimate for τ, i.e. $v = 1/\tau$, we finally estimate n

$$\begin{aligned} n &\sim 1 + (D-1)(\nu\hat{\rho} + (1-\nu)(1-\hat{\rho})) \\ &\sim 1 + (D-1)\left(\tfrac{1}{2} - (1-2\nu)\left(1 + \tfrac{1}{8}\nu\beta D\tau\right)\right) . \end{aligned} \tag{12}$$

which reduces to $n = 1 + \frac{1}{2}(D-1)$ for flat infection fronts ($\kappa = 0$), which again matches our initial naive expectation.

In a last step we can finally estimate n for low infection levels in the population, if ϵD interactions are long-range interactions, and therefore $n \sim 1$ for the nodes that get infected by a long-range link. The average n simply becomes

$$n = 1 + \frac{1-\epsilon}{2}(D-1) , \tag{13}$$

and therefore

$$D_c = 1 + \frac{2}{rd(1+\epsilon)} . \tag{14}$$

This formula estimates the critical degree as a function of the model parameters, r, d, and ϵ. We compare the theoretical predictions of D_c to the critical degrees from the simulations in Tab. 1 in the main text. The predictions nicely reproduce the qualitative behaviour of the parameter dependence of the critical connectivity D_c on ϵ, d, and r. The used second-order approximation is likely to systematically underestimate the spreading velocity, i.e. overestimate the number of infected in active regions of the network, which in turn leads to overestimation of D_c. This overestimation explains the deviations of theoretical and simulation results in Tab. 1. Also finite size effects in the simulation may add slightly to the deviations.

Text S7: A note on non-pharmaceutical interventions in relation to the model

The main purpose of the paper is on the explanation of the fact that many countries experience extended periods of linear infection curves. However, there are potential implications of that mechanism and the corresponding model insights in terms of non-pharmaceutical interventions (NPIs) that we discuss here.

Meaning of model parameters

There are obvious connection points between NPIs and the parameters in the model. To find out how they play out, one needs to carefully state the meaning of the three compartments susceptible (S), infectious(I), and recovered (R) as well as the parameters as we use them in the presented framework.

S remains to be the susceptible population, as in the usual SIR model. I is the *"free"* infectious population that is either aware or unaware (asymptomatic) of

its infectiousness. Both are in the position to infect others. The difference is that asymptomatic often go unnoticed and do not get tested. R includes the recovered who obtained immunity, the infected that have been isolated and can no longer infect others, and the fatalities.

The (average) time, d, of individuals being infectious, as used here, is not necessarily the time to recovery but a weighted average of the time to recovery and the time infectious individuals need to get detected and isolated. It is the average time infected people can infect others in their contact network. Therefore, d also depends on other factors such as the amount of successful infection contact-tracing that allows us to isolate infected persons earlier. With contact-tracing in mind, the parameter d can be used to model its effects. Effective contact-tracing will reduce d. The effect on the critical degree with reduced d can be read off in Table 1 for example. Unfortunately it is not possible at this stage to state how different types of contact-tracing and quarantine strategies effect d. The literature on the subject is presently still highly inconclusive.

Similarly, the average connectivity, D, as used here, does not simply include *all* interaction events of a person (maybe under given social distancing and mobility restrictions), but only those *"risky"* interactions, where transmissions are actually possible. In the context of COVID-19 these are interactions with close proximity, possible exchange of droplets, or exposure times of at least several minutes. It is these *"risky"* interactions where then the daily infection probability, r, applies as the *transmission probability.* In that sense, r quantifies the average "riskiness" of the *"risky"* interactions, whatever the term risky may include in particular. The question of what constitutes the actually relevant contact network presents a – maybe "the" – key question in the current pandemic. It is a tremendously difficult question, as the definition of the network depends on the nature of the epidemic. The assessment what this network should be is the topic of much ongoing work, that builds on pioneering work on the various types of actual temporal social contact networks [2, 5, 3, 4].

The transition to the known exponential behavior happens exactly when D becomes larger than the critical connectivity, D_c, and therefore we are confronted with a low-connectivity phenomenon, that can be the result of various NPIs that have been implemented across the world.

Relation of parameters and NPIs

The model parameter r is a biological constant, and can hardly be changed by NPIs. The other parameters can. For a functional understanding of how the change of these parameters affects the critical degree D_c, the relation, Eq. (14) might be helpful, $D_c \sim 1 + 2/(rd(1+\epsilon))$. The formula describes the general behavior found in the computational results that is shown in Table 1 in the main text.

Change of d: As mentioned, contact-tracing strategies and quarantine measures effectively reduce d. Many countries also implemented emergency telephone numbers, where people with symptoms called, and were asked to stay home until a negative test result was obtained. Also these cases were removed much earlier from the system, and effectively reduced d.

Change of ϵ: The role of NPIs, such as the switch to home-office and strict lockdowns (including closing work locations), effectively reduces ϵ and through it affects the critical degree. In the case of $\epsilon = 0$, in the spirit of the present model, all e.g. work-related contacts would be eliminated, contacts would essentially be reduced to interactions within households. Again, the effects of reducing ϵ can be understood from Eq. (14) and seen in Table 1. For example for the parameters as in the main text $r = 0.015$, $d = 14$, and $\epsilon = 0.3$ we get $D_c \sim 8.3$. Setting $\epsilon = 0$ we increase the critical degree by about 2, $D_c \sim 10.5$.

Change of D: As mentioned before, the social network topology parameter D is crucial. It depends strongly on implemented NPIs, which generally aim to reduce the average degree in the population. It also depends on the level of compliance in the population. It is beyond the scope of this paper, however, to know what that dependence would be exactly. Since several hundred different NPIs were implemented by most countries in various combinations and different points in time this would be a tremendous task. The much easier task to tease out the effective R from NPI data sets (as e.g. [7]) is already relatively hard to accomplish [8].

Given the implemented NPIs, and given that one observes the linear growth patterns this means that, effectively, in many countries the degree is below D_c. Depending on the country the (quasi constant) number of daily cases can be large (e.g. US) or small (e.g. Germany) but it is there. It does not vanish. In that sense the current non-pharmaceutical interventions have been insufficient. What would be needed for a full suppression of the epidemic is to bring the number of daily infections down to 0 for a time larger than d. This has been the strategy in China, but - given that they also lifted mobility and travel restrictions - also there they have not yet achieved full suppression.

Effect of timing of interventions

Can the model be used to estimate the impact of the time triggering interventions? Meaning to estimate the importance of timing of a given measure. We show that the model can indeed be used to demonstrate the relevance of timing of NPIs.

We proceed as follows. First note that the main purpose of the paper is to understand the origin of the linear infection curves. The result is that below a critical contact density one must expect extended periods of linear growth. However, this does not tell us what the linear increase actually is. Since we were

not able to compute the daily number of infected analytically as a function of the parameters, we must rely on simulations.

In SI Fig. 6 we show the same infection curves of the US (a) and Austria (b) as in Fig. 4 in the main text (blue lines). We show two scenarios. First we suppose that the US would have introduced measures on the date 0.1% of its population was tested positive. Suppose that the NPI impact of those would have been as strong as those implemented in Austria (when it faced 0.1% of the population infected). This means that for the US we switch the contact network from average degree $D = 5$ to $D = 2.5$ and the rewiring probability from $\epsilon = 0.3$ to $\epsilon = 0$. Transmission rate $r = 0.015$ and $d = 14$ days are kept as in the main text. The result is that the NPIs would have almost halved the number of infected until the first week into May 2020, see SI Fig. 6 (a).

The second scenario is to demonstrate the impact of timing of implementation of NPIs. We assume that the same NPI that Austria implemented was implemented 10 days later. Again the NPI consisted in switching $D = 5$ to $D = 2.5$ and the rewiring probability from $\epsilon = 0.3$ to $\epsilon = 0$, and keeping the other parameters as in (a). The result shows that timing matters: would the measures have been delayed by ten days, this would have resulted in almost 30% more cases.

This might be an explanation why countries that acted strongly in the beginning of the pandemic, are usually much better of than those that did not, both in term of infected and fatalities.

Note that this kind of dynamics can of course be modeled with the SIR model and derivates, however, these will of course not take into account the low-degree effects correctly.

Finally, we can also make an analytical statement on how D_c can be related to the time of implementation of NPIs. earlier or later measures. In the course of the analytical derivation of D_c in Section S6 an interesting detail can be observed: as the number of susceptible nodes in the population decrease, the critical degree D_c rises. In other words, the earlier a measure is implemented, the slower susceptibles diminish, and the longer D_c remains low. If this effect bears any practical implications remains to be seen. In Section S6 we discuss this for random networks (which model the case of no mobility restrictions) and find that this effect is also responsible for shifts in herd immunity levels, i.e. the fraction of nodes that will not be infected during the epidemic. Note also that in the model, herd immunity is reached when the decreasing fraction of susceptibles increases the critical D_c above the actual degree D in the population.

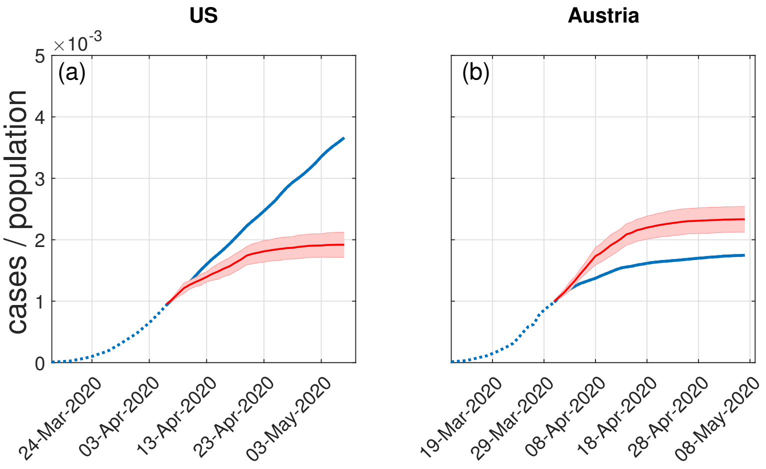

Figure 6: Demonstrating (a) the order of magnitude of the impact of NPIs, and (b) the importance of their timing (b). We show two scenarios. (a) suppose the US would have introduced NPIs that reduced the average degree in the contact network from $D = 5$ to $D = 2.5$ and $\epsilon = 0.3$ to $\epsilon = 0$. The number of infected turn out to be almost half the number of infected up to the first week in May 2020, (b) If the same NPI that Austria implemented was implemented 10 days later by switching $D = 5$ to $D = 2.5$ and the from $\epsilon = 0.3$ to $\epsilon = 0$ this would have resulted in up to 30% more cases. Compare to Fig. 4 in the main text.

Limitation for practical use

One has to bear in mind that one is dealing with a stochastic process with a reproduction number in a critical regime with a value of one, meaning that the process is *critical* and therefore likely to exhibit local and/or temporal fluctuations in the connectivity D, d, and ϵ. The situation is further complicated by the non-linear nature of SIR type models and the particular topologies of the networks - which may play out strongly in low-density regimes as the one studied here.

Running the models with the same set of parameters for many times shows these uncertainties clearly: In the linear regime, infections die out with a non-vanishing probability. Moreover, by fluctuating around more or less constant average reproduction numbers, they still vary from run to run. This implies that individual countries, even if they implemented the same measures at the same time, may still vary substantially in terms of their infection trajectories. Total infection numbers that can easily differ by a factor two or even more after a 100 days.

Using more realistic social contact networks

The two network models used in this paper (Poissonian small-world and normal small-world) are inspired by the fact that social networks are structured in a way that many small communities are linked among each other in very heterogeneous ways. Small communities, such as families are linked by the individuals that have contacts with members of both, be it at work, school, clubs, etc. In the model we used, many overlapping small communities are additionally linked to each other through "short cuts". These are modeled as long-range links. Their fraction w.r.t. to short-range-community links is given by the parameter ϵ.

The proposed (Poissonian) small-world networks could be over-simplistic, and might introduce biases and unrealistic features. To see if the described infection mechanisms are still present in a more realistic setting, we used a number of real social networks in different contexts. These are temporal contact networks that were analyzed in detail in [2, 3, 4], and that are made available on the *sociopatterns* homepage [5]. These networks contain practically a large number of traced contacts between people in workplaces, primary and high schools, hospitals and homes, between 1 and 3 weeks of observation time.

From these temporal networks we derive weighted links between all persons that quantify the number of contacts between two people over the time the sample extends. The resulting networks are dense, practically everybody has been in contact with a large fraction of others, usually through very few contacts – most of them originating from random encounters. To find repeated contacts that indicate sufficiently strong social contacts, we simply threshold these weighted networks by removing all links from the network that have a weight below a threshold, θ. We show the obtained networks in Fig. 7 (a)-(c) by setting $\theta = 100$, 200, and 500. With this filtering they have an average degree of $D = 31.5$, 10.7, and 4.1, and the number of nodes is 213, 209, and 139, respectively.

We now use these networks to run the algorithm used in the main text, with $r = 0.02$, and $d = 14$ and create the infection curves, shown in Fig. 7 (d). Individual realizations are shown. For the dense network that is clearly above D_c, the infection curve (blue) follows the SIR model closely (green dotted line), but does not quite reach the SIR plateau. Similarly, for the network with $D = 10.7$ that is closer to D_c the situation is similar (red). What is interesting is that the case for the network with the highest threshold $D = 4.1$ that is likely to be below D_c, after an initial phase following the SIR curve, we find the typical linear growth for an extended period of time (green).

In Fig. 7 (a)-(c) the colours of nodes show the nodes' states after 100 timesteps. Green is susceptible and red is recovered. For the dense network (a) the core of the network got infected, just a few peripheral nodes were never infected. For (b) the situation is similar. For the sparse version of the network, it is clear that a few clusters got infected. That is the typical pattern that is indeed observed in the COVID-19 pandemics, see e.g. [1]. Note that in other realizations of the

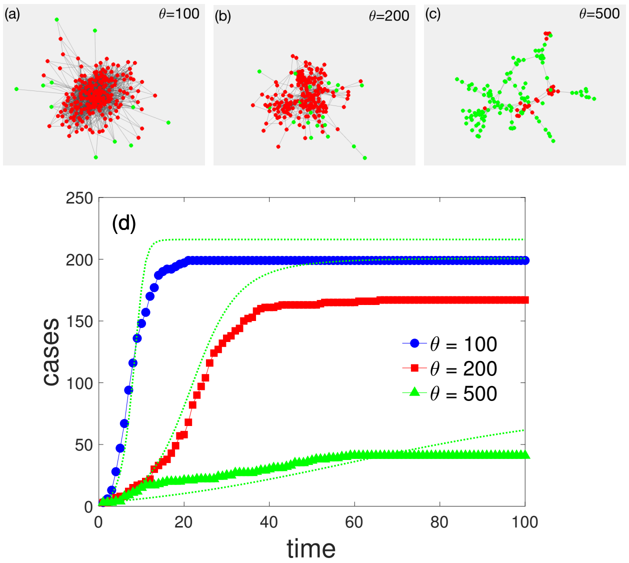

Figure 7: Social temporal contact networks of people at a workplace (InVS15) [2] thresholded according to the number of contacts. In (a) all links are shown that connect two persons with more than $\theta = 100$ encounters, (b) and (c) show the cases for $\theta = 200$ and $\theta = 500$. As the networks get sparser, clear clustered structure between "strong" contacts becomes visible. In (d) we show corresponding realizations of infection curves with 3 initially infected. The two dense networks (with $D > D_c$) show SIR like behavior (green dashed line), while the infection curves for the low density network ($D < D_c$) shows the typical linear increase over an extended time. In (a)-(c) the green means susceptible and red recovered after the simulation converged. In (c) it is clearly visible that some highly connected clusters were infected, which is a typical feature in the COVID-1 pandemic.

infection curves, slightly different patterns can occur due to the very limited size of the networks, and the presence of strong clustering.

For these reasons we conclude that also for more realistic contact networks the same mechanism is present as described in the main text, and the reasoning why linear infection curves emerge remains fully valid. The fact that for a reasonably low-density network the typical spreading patterns through infection-clusters gives further evidence for the consistency of the approach.

We close by noting that the actual contact network structures in combination of the infection characteristics of different epidemics are highly relevant [6, 2]. The nature of these temporal networks becomes clear only recently [2, 3, 4, 6] Many of the networks studied are presently subnetworks, the "entire" social network o the globe, is still beyond scope.

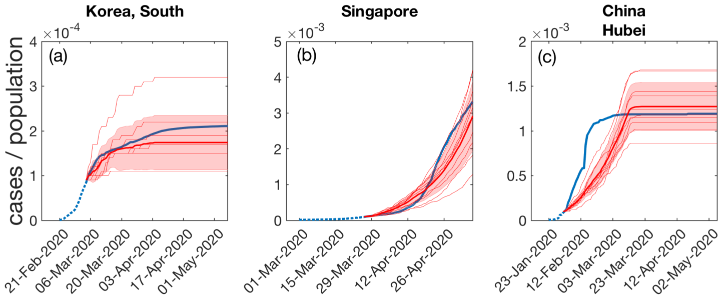

Figure 8: Comparison of infection curves (blue) of the three countries, Korea (a), Singapore (b), and the Chinese province of Hubei(c) that followed different paths through the initial phases of the pandemic. Model results for infection curves under different ways of implementing NPIs (that mimic the behavior of the countries) are shown as thin red curves. Their average and standard deviation is the thick line and the shaded area, respectively. Clearly, the model results follow the qualitative behavior nicely.

Note on recent developments later in the pandemics

By July 2020 many countries have (at least partially) taken back many NPIs, resulting in a (seemingly exponential) resurgence of daily infections. In some countries the highest daily rates have been observed in July, higher than in March and April, where many countries experienced their first maximum of daily cases.

The return of more infections is maybe not yet the "second wave", but just the logical response of reduction of social distancing and increase of D, d, and ϵ.

Various degrees of how NPIs have been taken back and sometimes re-implemented led to a very diverse range of infection curves in the meantime. In the beginning of the pandemics the picture was much more homogeneous and allowed to observe the linear curves in a clear way. Here also the dimensional effects mentioned above may play a role, if social distancing is insufficient to keep the local contact network from being essentially one-dimensional, such as spanning trees or circles as we used in the model. If the contact network starts to resemble a two dimensional lattice, then also powers of $1 < \alpha \leq 2$ are conceivable.

Text S8: Case study of 3 Asian countries with different strategies

We compare three different scenarios of implementing NPIs in the model that resemble actions and situations of three actual countries. We compare south

Korea, Singapore, and the Chinese province of Hubai. In SI Fig. 8 we show their infection curves and the results of three implemented strategies of the model. We try to roughly mimic NPIs implemented by these countries in the early phase of the pandemic and compare the outcome (red) with the actual infection curves that are shown in blue. The same basic strategy as in Fig. 4 in the main text is followed. We show 10 individual trajectories, to see that the qualitative behavior of trajectories better (thin red lines); the thick red line represents the average over these trajectories, the shaded region indicate the errorbars (std).

Korea: Korea was very early in imposing strong measures in quarantining contact persons and installing contact-tracing (both Feb 4, 2020), health checks at airports, and education of how to do self-quarantine (both Feb 12, 2020), closing air traffic (Feb 22, 2020) social distancing including closing of educational institutions (Feb 24, 2020), and requiring usage of masks (Feb 28, 2020). For the dates implemented and an overview of the NPIs, see for example http://covid19-interventions.com.

After a moderate exponential start, since about mid-March infection numbers were entering a linear regime, that ended about mid April. This was followed with very low numbers of cases with the exception of a few bursts that were brought under control quickly.

We model the infection dynamics by starting after 0.01% of the population is infected, with a contact network ($N = 100,000$) with $D = 7$ and $\epsilon = 0.3$. After 3 days measures are imposed that shift to network with $D = 4$ and $\epsilon = 0$, mimicking the reduction of work contacts and long term links. We used the same transmission rate $r = 0.015$ and $d = 14$ days. This is a slightly denser (Asian) contact network than the ones used for the US and Austria in the main text. After a few days a somewhat linear regime starts to emerge, that finally flattens off almost completely. The results are seen in SI Fig. 8 (a).

If we reduce d to, say 10, at the time of the change to the new network, we obtain slightly worse results. This means that the observed effects seen here are network-driven. The simulation-averages over 10 independent trajectories are shown in the thick line. The average only captures the situation partly, mainly because of the mentioned instabilities and the resulting confidence intervals. Most individual trajectories do follow the real trajectory qualitatively, however.

Note that we do not perform a systematic scan over parameter space. It would be impossible to do since the creation of a single Poissonian small-work network with 100.000 nodes can take up to an hour, depending on D and ϵ.

Singapore: Singapore was one of the earliest nations besides China to employ strict measures from Jan 20 on. New NPIs were introduced almost daily until Feb 12, including quarantine, health checks at airports, quarantine for contact persons, case isolation, border restrictions, closing of educational institutions. For details, see http://covid19-interventions.com. Initially Singapore was extremely successful and among the countries with the lowest numbers of daily

cases until first cases in workers' communities appeared, which got out of control and lead to an exponential phase beginning approximately in the second week of April, similar to the ones experienced in other countries earlier. See SI Fig. 8 (b).

We start with a society that had implemented NPIs and has already switched to a low-degree network with $D = 3$ and $\epsilon = 0$. Since people are immediately and effectively quarantined, we set $d = 5$ days. As before, we use $r = 0.015$.

To model the spread within the workers' community, we assume that now the the virus hits a different population (workers' communities within Singapore) that live together much more densely, less separated and who are less monitored by the state institutions. We thus switch to a network with higher density, $D = 7$ and $\epsilon = 0.3$ And we increase to $d = 14$ days and assume a slightly higher transmission rate of $r = 0.020$. Again, we find the correct qualitative behavior, seen in See SI Fig. 8 (b). After a slow linear growth in the beginning an exponential phase hits. We crossed the critical D_c.

Hubei: The Chinese province of Hubei was affected by extremely drastic measures imposed early on. On Jan 23 the city of Wuhan was completely isolated by air, bus and train traffic. Small and mass gatherings were forbidden, mandatory measuring of body temperature was implemented, all but pharmacies and supermarkets were closed, movement restrictions including usage of private cars, and many more, were all in place by Feb 17. After an initial exponential phase, the situation in Hubei was brought under control, there are practically no new cases from the beginning of March on, see SI Fig. 8 (c). Thus also no linear growth.

We model the initial situation close to critical parameters $D = 7$ and $\epsilon = 0.3$. We use $d = 14$ days and $r = 0.015$. End of Feb the government switches to a new network by imposing even harsher NPIs. In the model we account for that by switching to a network with $D = 4$ and $\epsilon = 0.1$, and set $d = 4$, assuming very effective quarantine measures.

The model does what is expected. The exponential growth stops quickly and no more cases appear. The fact that the different trajectories reach different maxima is attributed to the fact that – naturally – stopping the exponential growth leads to a wide variability in the final numbers. Details in the network and the initial configuration of infected do matter.

The fact that we underestimate the initial exponential growth is that there seems to be a strange behavior in the infection curve itself, that might be due to increased testing and testing rates beginning Feb until mid Feb. One might have to use a higher D. It is,however, not the point here to perfectly match the curves, but to show that the model is able to pick up the essential features of NPIs that were actually implemented.

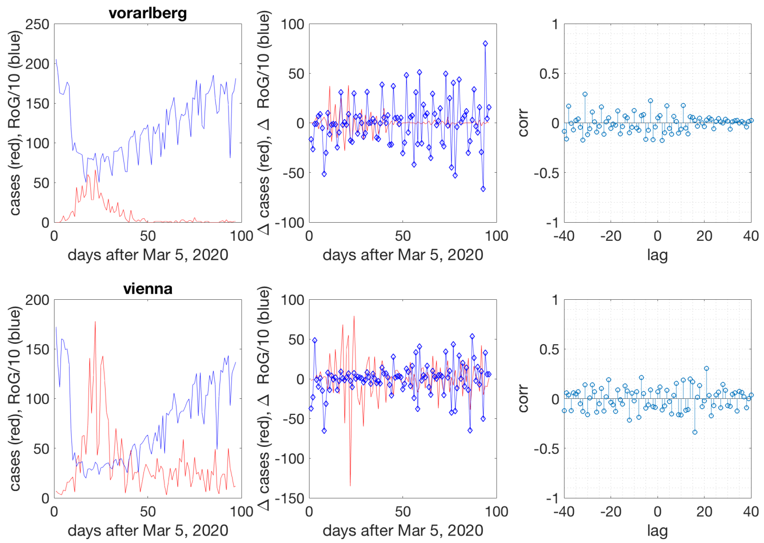

Figure 9: (Left) Measure of mobility,, radius of gyration (RoG) in the rural state Vorarlberg (top) and Vienna (bottom), in blue. The daily cases of COVID-19 infections are show over the same time, March 5 - June 10, 2020. (center) Fluctuations as defined as the first differences, of the same timeseries. Clearly, they are stationary, and a cross-correlation analysis is justified. (right) Cross-correlation values as a function of lags (mobility leads cases). No significant correlations are present, except a visible 7-day periodicity that stems from the strong weekend effects in mobility. This pattern vanishes if weekends are removed from the timeseries.

Text S9: Excluding mobility as a confounder

We want to test for the possibility that the reduction of mobility that was observed in many countries to various degrees, could serve as an explanation for R fluctuating around the value of 1. There are indications that mobility could very well be a relevant confounder, see e.g. the review article [9].

To do so, we follow the following simple, but effective strategy. As a measure of mobility, we use averaged mobility data (radius of gyration derived from cell phone data) of all states of Austria[1].

[1] We have access to mobility data based on mobile phone data through a Austrian telephone (4.5 Million per day). The provider provides us with localization information for each cell-id, which is based on the centroid of the network coverage simulation. The data is annonymized, any identifiers are hashed every 24 hours with a changing salt, only cell-id based localization is used to enhance the privacy of the subscribers. Only aggregate and k-anonymized statistics are available. We adhere to the GSMA guidelines [10] with regards to data privacy handling in the case of COVID-19 as well as the law of the local jurisdiction (Austria). From this data

For comparison, we present the RoG(t) for the smallest rural state (Vorarlberg, population 397,094) and the city of Vienna (population 1,911,728) in Fig. 9 (blue curves, left panels). Time runs from March 5 to June 10. There was a nation-wide lockdown in Austria on March 16, wich is clearly visible in the mobility patterns. Mobility gains momentum slowly over the following weeks, almost reaching pre-lockdown levels toward the end of the timeseries. In the same panels the daily cases $C(t)$ in the respective states are shown (red).

We first test if the fluctuations in mobility ($\Delta RoG(t) = RoG(t) - RoG(t-1)$), where t are days, lead the fluctuations in the cases, $\Delta C(t) = C(t) - C(t-1)$. These difference-timeseries are clearly stationary, (center panels) and can be used in a cross-correlation analysis. In the right panels we see the (normalized) correlations as a function of the lags. only very minor correlations are obtained, which are at a respective distance from each other of 7 days, which obviously capture weekend effects that are strongly visible in the raw data (left panels). To exclude these, we performed the same procedure with weekends taken out of the timeseries. The 7-day correlations vanish, no significant correlations are observable. The largest correlation value appears at -31, which is excluded to be of any potential significance.

To test directly the potential effects of mobility, we compute the cross correlation of $\Delta RoG(t)$ and the fluctuations in R_{eff} (i.e. $\Delta R_{\text{eff}}(t) = R_{\text{eff}}(t) - R_{\text{eff}}(t-1)$), which we obtained from as the official data of the *Austrian Agency for Health and Food Safety.* For data and information, see [11] (in German). We show the results in Fig. 10. Again, we find no hint for any correlation. We also performed a cross correlation analysis of $\Delta RoG(t)$ and R_{eff}. We are aware that since R_{eff} is not stationary, high correlation values would be questionable and hard to interpret. However, again, we only find tiny correlation-values of about 0.05.

We can therefore safely exclude the possibility for a direct lead-lag influence of mobility on R_{eff}, which also practically excludes out the possibility that mobility effects drive R towards 1.

References

[1] Kupferschmidt K. (2020) Case clustering emerges as key pandemic puzzle. *Science* 368, 6493:808-809.

[2] Genois M., Barrat A. (2018) Can co-location be used as a proxy for face-to-face contacts? *EPJ Data Science* 7:11.

we compute the median radius of gyration (RoG) as the square root of the time weighted mean of the squared distances of the device localizations to the daily centroid. It captures the amount of movement in a time weighted manner and has the dimension of length. We compute the RoG as a measure of daily movement of every device located in any state of Austria. We then take the median over all RoGs on every day, from March 5, 2020 on.

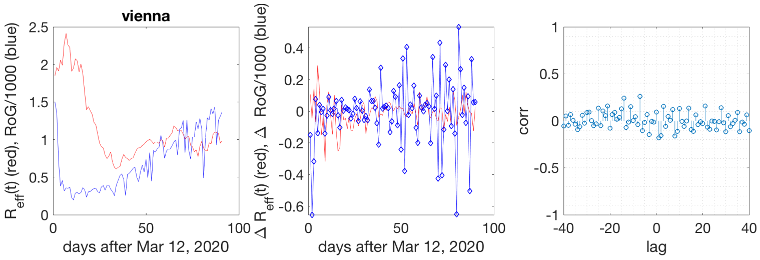

Figure 10: Same as previous figure, however with the daily cases replaced by the effective reproduction number, R_{eff} for the State of Vienna. Again, no signs for significant correlations are present.

[3] Sekara V, Stopczynski A, and Lehmann S. (2016) The fundamental structures of dynamic social networks. *Proceedings of the National Academy of Sciences USA* 113 (35):9977-9982.

[4] Stopczynski A., Sekara V., Sapiezynski P., Cuttone A., Madsen M.M., Larsen J.E., et al. (2014) Measuring Large-Scale Social Networks with High Resolution. *PLoS ONE* 9(4):e95978.

[5] SocioPatterns, interdisciplinary research collaboration. http://www.sociopatterns.org/datasets/

[6] Stopczynski A., Pentland A., Lehmann S. (2015) Physical Proximity and Spreading in Dynamic Social Networks, *arXiv* 1509.06530.

[7] Desvars-Larrive A. et al. A structured open dataset of government interventions in response to COVID-19. *medRxiv* 2020.05.04.20090498.

[8] Haug N., Geyrhofer L., Londei A., Dervic E., Desvars-Larrive A., Loreto V., Pinior B., Thurner S., Klimek P., Ranking the effectiveness of worldwide COVID-19 government interventions. *medRxiv* 2020.07.06.20147199.

[9] Nuria, O. et al. (2020) Mobile phone data for informing public health actions across the COVID-19 pandemic life cycle. *Science Advances* 6:eabc0764.

[10] GSMA. The GSMA COVID-19 Privacy Guidelines. Tech. rep. April. GSMA, 2020. https://www.gsma.com/publicpolicy/wp-content/uploads/2020/04/The-GSMA-COVID-19-Privacy-Guidelines.pdf

[11] https://www.ages.at/en/wissen-aktuell/publikationen/epidemiologische-parameter-des-covid19-ausbruchs-oesterreich-2020/
